# Green Routes for the Production of Enantiopure Benzylisoquinoline Alkaloids

**DOI:** 10.3390/ijms18112464

**Published:** 2017-11-20

**Authors:** Francesca Ghirga, Alessandra Bonamore, Lorenzo Calisti, Ilaria D’Acquarica, Mattia Mori, Bruno Botta, Alberto Boffi, Alberto Macone

**Affiliations:** 1 Center for Life Nano Science@Sapienza, Istituto Italiano di Tecnologia, Viale Regina Elena 291, 00161 Rome, Italy; francesca.ghirga@uniroma1.it (F.G.); mattia.mori@iit.it (M.M.); 2 Deaprtment of Biochemical Sciences “A.Rossi Fanelli”, Sapienza University of Rome, Pizzale Aldo Moro 5, 00185 Rome, Italy; Lorenzo.calisti@uniroma1.it (L.C.); alberto.boffi@uniroma1.it (A.B.); 3 Department of Chemistry and Technology of Drugs, Sapienza University of Rome, Pizzale Aldo Moro 5, 00185 Rome, Italy; bruno.botta@uniroma1.it

**Keywords:** alkaloids, benzylisoquinoline alkaloids, green synthesis, recombinant enzymes, microbial factories

## Abstract

Benzylisoquinoline alkaloids (BIAs) are among the most important plant secondary metabolites, in that they include a number of biologically active substances widely employed as pharmaceuticals. Isolation of BIAs from their natural sources is an expensive and time-consuming procedure as they accumulate in very low levels in plant. Moreover, total synthesis is challenging due to the presence of stereogenic centers. In view of these considerations, green and scalable methods for BIA synthesis using fully enzymatic approaches are getting more and more attention. The aim of this paper is to review fully enzymatic strategies for producing the benzylisoquinoline central precursor, (*S*)-norcoclaurine and its derivatives. Specifically, we will detail the current status of synthesis of BIAs in microbial hosts as well as using isolated and recombinant enzymes.

## 1. Introduction

Benzylisoquinoline alkaloids (BIAs) are a nitrogen-containing group of plant secondary metabolites with a centenary history of investigation [1]. The interest in the wide variety of BIA structures is paramount, due to their well-established and long-standing pharmacological profiles [2]. The most covered therapeutic areas by BIAs are analgesics [3,4], antimicrobial [5], antimalarial [6,7], anti-HIV [7,8,9], and anticancer agents [10,11,12], whereas new areas are being explored more recently for BIAs, such as their employment as cholesterol-lowering and protecting agents against the atherosclerotic disease [13,14,15,16] and as signaling suppressors in colon cancer cells [17].

In 2010, with the aim of providing comprehensive information about both the source and the medicinal properties of BIAs, there was created an *ad hoc* database of benzylisoquinolines compounds, called BIAdb, helpful for all those who are working in the field of drug discovery, to explore the pharmacological potential of BIAs [18]. One of the most important features of BIAdb is that it provides information about 627 plant species as a source of benzylisoquinoline and 114 biological activities shown by these compounds. A lot of tools have been integrated in the database, which help users in discovering the full potential of the program itself.

**Figure 1:**
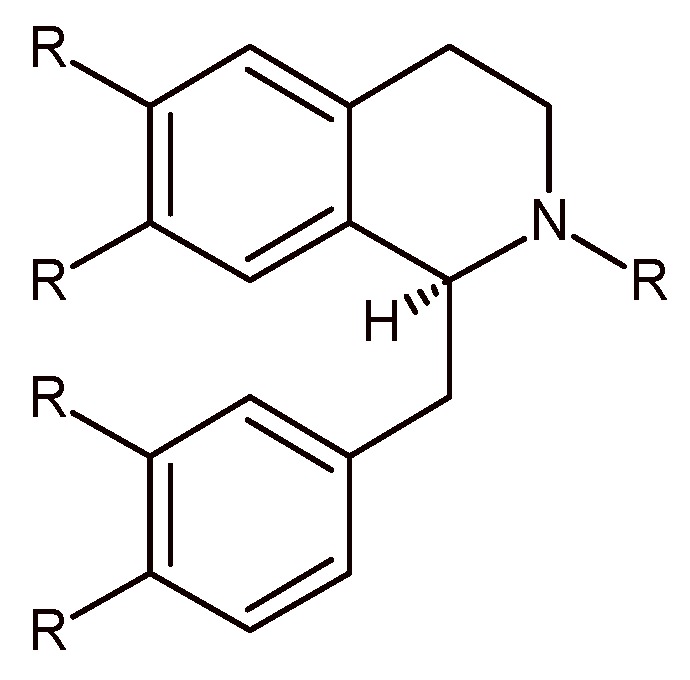
The benzylisoquinoline skeleton (R = H, OH, OMe).

The general benzylisoquinoline skeleton (Figure 1) can be mostly synthesized (with few exceptions) by a cyclization reaction, namely, the Pictet-Spengler (P-S) reaction, which has been the subject of a huge number of review articles [19,20,21,22] and in 2011 has entered its second century of life [23,24]. Nowadays, the P-S reaction is still one of the most employed synthetic tools not only to yield tetrahydroisoquinolines (THIQs) and tetrahydro-β-carbolines (THBCs), but also to build novel scaffolds for structure-activity relationship studies and/or for combinatorial libraries for drug discovery programs.

**Scheme 1:**
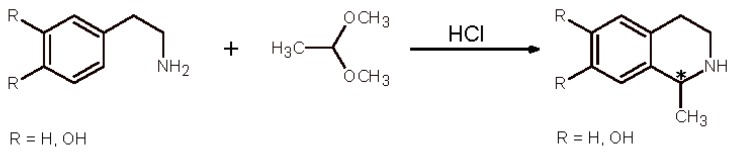
First appearance of the Pictet-Spengler (P-S) reaction. The asterisk denotes the chirality centre at C-1.

The P-S reaction is essentially an electrophilic aromatic substitution and can be described as a modification of the Mannich reaction. The name comes from the chemists Pictet and Spengler, who synthesized 1-methyl-1,2,3,4-tetrahydroisoquinoline (Scheme 1) in 1911, by cyclocondensation of β-phenethylamine with acetaldehyde dimethyl acetal catalyzed by HCl [25]. With the exception of formaldehyde, any aldehyde which is submitted to the P-S reaction creates a new chirality centre at the C-1 carbon atom of the THIQ skeleton; this is the reason why the stereoselectivity of the reaction in the synthesis of isoquinoline alkaloids has been an ancient competition for organic chemists.

**Scheme 2:**
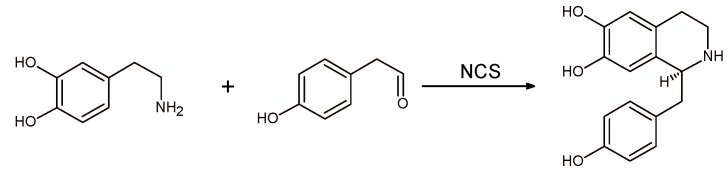
Biosynthetic pathway for (*S*)-norcoclaurine alkaloid catalyzed by the norcoclaurine synthase (NCS) enzyme.

Despite the huge structural diversity, BIAs have the same biosynthetic precursor, namely (*S*)-norcoclaurine (Scheme 2), which is formed by condensation of dopamine and 4-hydroxyphenylacetaldehyde (4-HPAA), with an excellent stereoselectivity [26,27]. The enzyme involved in the biosynthesis is the norcoclaurine synthase (NCS; EC 4.2.1.78), which can be defined as a “Pictet-Spenglerase”. One of the most fascinating features of BIAs, in fact, is that they are synthesized in the plant as single enantiomers, as usually happens for natural products, even though this is not always true [28]. Moreover, there are cases where enantiomerically opposite metabolites are produced by different genera or species, or even they have been isolated from different portions of the same plant [27].

**Scheme 3:**
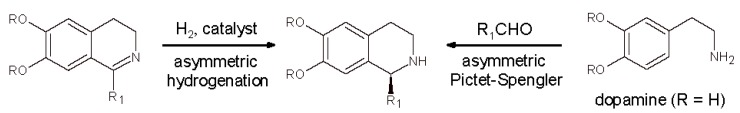
The two general enantioselective methods for the preparation of 1-substituted tetrahydroisoquinolines (THIQs) skeleton (R_1_ = alkyl or aryl).

The major strategies that have been developed so far to synthesize the biologically active (*S*)-enantiomer of 1-substituted THIQ alkaloids are the following two [29]: (i) the Bischler-Napieralski cyclization/reduction sequence of dihydroisoquinoline intermediates; and (ii) the P-S condensation of β-arylethylamines such as dopamine with a variety of aldehydes (Scheme 3).

In the first case, it is the reduction step that governs the introduction of the chirality centre at the C-1 carbon atom; therefore, chiral hydride reducing agents must be employed, or else the hydrogenation must be carried out in the presence of chiral catalysts. In the last case, the absolute configuration of the catalyst is a fundamental tool to prepare any of the desired alkaloid enantiomers.

In the second method, the chirality centre at C-1 is formed during the ring closure step, in a one-pot fashion. As a result, a properly added chiral auxiliary must be capable of transferring its chirality to either the β-arylethylamine or the aldehyde, and this means that a diastereoselective synthesis occurs.

**Scheme 4:**
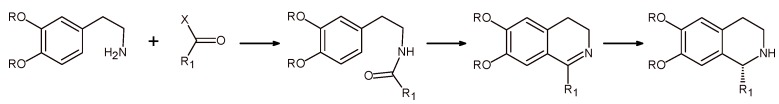
Bischler-Napieralski cyclization/reduction sequence for the synthesis of chiral isoquinoline alkaloids.

The Bischler-Napieralski cyclization/reduction sequence (Scheme 4) has been the most used synthetic approach for the asymmetric production of THIQs [29]. According to it, a β-arylethylamide undergoes a cyclization to 1-substituted dihydroisoquinoline or a corresponding dihydroisoquinolinium salt, followed by a reduction to tetrahydroisoquinoline. Transition metals such as ruthenium (Ru), rhodium (Rh), and iridium (Ir) have been successfully employed as chiral catalysts in the asymmetric hydrogenation of a variety of dihydroisoquinolines, resulting in high enantiomeric excess (*ee*) values with a growing number of substrates [30,31,32].

**Scheme 5:**
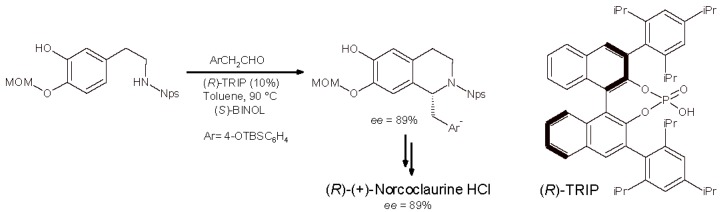
Asymmetric P-S reaction of 3-hydroxy-4-MOM-phenylethylamine as *O*-nitrophenylsulfenyl derivative (Nps) with arylacetaldehydes. (MOM = methoxymethyl; iPr = *iso*propyl).

In 2015, a general approach has been described to the enantioselective synthesis of 1-benzyl-1,2,3,4-THIQs based on an asymmetric P-S reaction catalyzed by BINOL-phosphoric acid [(*R*)-TRIP] [33]. The novelty of the method has been the introduction of a moderately strong electron-withdrawing group on the nitrogen atom of the phenylethylamine, i.e., the *O*-nitrophenylsulfenyl (Nps) substituent (Scheme 5). Many biologically active alkaloids have been synthesized thanks to this approach, such as (*R*)-crispine, (*R*)-coclaurine, (*R*)-laudanosine, and (*R*)-calycotomine. (*R*)-Norcoclaurine was obtained as well as its hydrochloride salt (HCl), with 89% *ee*, and the same synthetic protocol with the (*S*)-TRIP catalyst might lead to the biologically relevant (*S*)-enantiomer.

Among the numerous synthetic efforts to easily access enantiomerically pure BIAs to be tested as novel drugs, the stereochemical feature of these alkaloids is currently being studied to assign the correct absolute configuration of some alkaloids isolated from different parts of a plant. To this purpose, the (*R*)- and (*S*)-diastereoisomers of norcoclaurine 4′-*O*-β-d-glucoside were synthesized [34] and their NMR and HPLC profiles were compared to unequivocally assign the stereochemistry of a previously isolated product [35].

**Scheme 6:**
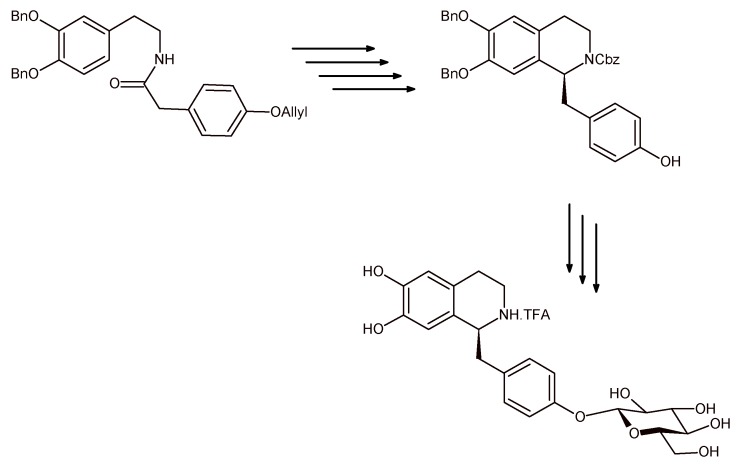
Synthetic pathway to (*S*)-norcoclaurine-4′-*O*-β-d-glucoside by enantioselective Bischler-Napieralski cyclization/reduction sequence and coupling with 2,3,4,6-tetra-*O*-acetyl-β-d-glucopyranosyl 2,2,2-trichlotoacetimidate. Each arrow of the design represents one step in the synthetic process.

Briefly, the Bischler-Napieralski cyclization/reduction sequence of a proper amide (Scheme 6) by employing RuCl [*R*,*R*-TsDPEN(*p*-cymene)] as the asymmetric catalyst yielded the (*S*)-*N*-Cbz-6,7-di-*O*-benzylnorcoclaurine, which, after the attachment of a glucosyl moiety to the *p*-hydroxybenzyl unit and subsequent deprotection gave the (*S*)-norcoclaurine-4′-*O*-β-d-glucoside as trifluoroacetate (TFA) salt in 93% yield and 95% diastereomeric excess.

Although many elegant syntheses of BIAs have been proposed, the development of more efficient synthetic routes is still a great challenge to the chemists due to the intricate molecular architecture of these molecules. Usually, chemical synthesis of alkaloids is complex due to the presence of multiple stereogenic centres and requires multistep procedures, many of which involve the use of protective groups resulting in moderate to poor yields.

As a matter of fact, one of the major limitations in the chemical synthesis of BIAs is the scale up of the process that, even if feasible, it still remains not economically convenient.

Moreover, direct extraction of alkaloids from plants is often impractical, as they are often produced in very small amounts by their natural source. For all these reasons, in this last decade new synthetic processes have been developed joining the expertise of chemists with the new tools emerging in the field of biotechnology [36]. These alternative pathways, involving microorganisms such as bacteria and yeast as well as purified enzymes, allow rapid and stereospecific production of large quantities of advanced intermediates, reducing at the same time waste products generated during the synthetic process, thus making the overall process green and environment friendly. These topics will be described in Section 2 and Section 3. 

## 2. Working with Living Organisms: Biotechnological Approaches in Benzylisoquinoline Alkaloids (BIA) Production

### 2.1. BIA Production in Plants

The growing interest in plant alkaloids and their use for therapeutic purposes has led to the development of different extraction methods. In fact, due to their complex chemical structures, when feasible BIAs are directly extracted from plants. However, very often these methods consist in expensive and time-consuming procedure and the isolation yields are generally too low. Moreover, the secondary metabolite patterns of plants grown at different times and places with different physical and chemical stimuli can vary greatly and this represents a major obstacle in using plants as a drug producing source. Hence, the production of plant derived drugs requires genetically uniformed monocultures grown in completely standardized conditions.

BIA metabolism involves many enzymes belonging to a small number of protein families, including *S*-adenosylmethionine (SAM)-dependent *N*- and *O*-methyltransferases, cytochromes P450, NADPH dependent dehydrogenases/reductases, FAD-dependent oxidoreductases, *O*-acetyltransferases, and *O*-demethylase [1].

All these enzymes can be the target of metabolic engineering both for functional studies and to improve BIAs production in plant. To this purpose, biotechnological approaches such as knock-out, knock-down, or overexpression of selected genes encoding for key enzymes involved in alkaloid metabolism have been employed. Overexpression of genes encoding for rate-limiting enzyme, for instance, can favour the accumulation of end products; introduction of new genes from different species can create novel products; RNAi, virus-induced gene silencing (VIGS) or clustered regularly interspaced short palindromic repeats associated with Cas9 endonuclease (CRISPR/Cas9) can decrease undesired compounds or can help to accumulate valuable intermediates.

Recently, it has been reported that opium poppy transformed with constitutively expressed gene for codeinone reductase, showed a higher content of morphinan alkaloid [37,38]. *Coptis japonica* was modified to improve alkaloid yields by overexpressing the 3-hydroxy-*N*-methylcoclaurine-4-*O*-methyltransferase (4-OMT) gene, one of the key enzymes in BIAs biosynthesis in this species [39,40]. Overexpression of the *Papaver somniferum* berberine bridge enzyme (BBE) in *Eschscholzia californica* led to the accumulation of the end products benzophenanthridine alkaloids compared to controls [41]. Overexpression of salutaridinol 7-*O*-acetyltransferase (SalAT), a key gene in morphinan alkaloids biosynthesis pathway, specifically resulted in increased morphinan alkaloid accumulation in transgenic hairy root lines from *Papaver bracteatum* [42,43].

**Scheme 7:**
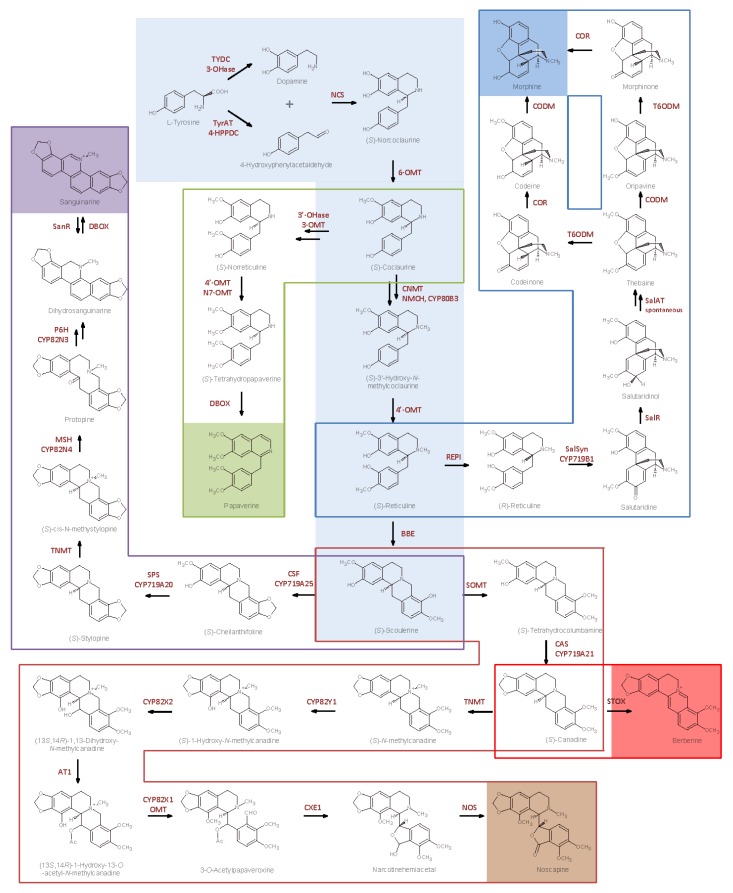
Main pathways for the biosynthesis of benzylisoquinoline alkaloids (BIAs). Light blue: pathway leading from Tyrosine to (*S*)-Scoulerine; green line: pathway leading from (*S*)-Coclaurine to Papaverine; blue line: pathway leading from (*S*)-Reticuline to Morphine; purple line: pathway leading from (*S*)-Scoulerine to Sanguinarine; brown line: pathway leading from (*S*)-Scoulerine to Noscapine; red line: pathway leading from (*S*)-Canadine to Berberine. The end-products of the metabolic pathways are in full color squares. The enzymes catalyzing each reaction step are reported in correspondence of the reaction arrow.

RNAi has been also used to create transgenic poppies displaying high levels of reticuline rather than the narcotic morphine [44]. After gene silencing, the intermediate (*S*)-reticuline, which is produced several steps upstream of codeinone (see Scheme 7), accumulated in transgenic plants at the expense of morphine, codeine, oripavine, and thebaine. Methylated derivatives of reticuline also accumulated.

VIGS has been used as a functional genomic tool to investigate the regulation of morphine biosynthesis by reducing the levels of enzymes catalyzing the last six steps in morphine biosynthetic pathway [45]. Even though the study was focused on validating the physiological role of biosynthetic genes involved in morphinan branch pathway, the increase of reticuline, salutaridine, thebaine, and codeine concentrations could make this approach suitable for the generation of plant biofactories (Scheme 7).

CRISPR/Cas9 system is a new tool in molecular biology which provides a number of applications in metabolic engineering and molecular farming, characterized by higher accuracy and precision compared to other well-known genome editing tools [46]. Very recently, CRISPR/Cas9 system has been employed to knock-out the 4′-OMT2 gene involved in the regulation of the biosynthesis of many BIAs including noscapine and morphine [47]. This study paves the way for further research aimed at addressing some issues connected to BIA biosynthesis.

Although plant engineering has been used to induce secondary metabolites accumulation [40,48,49,50], it is not easy to achieve the desired products because their biosynthesis are tightly regulated. For example, in most pathways, overexpression of one enzyme results in making subsequent reactions more limiting. In the same way, blocking the expression of a selected enzyme very often results in a lower concentration of downstream metabolites, but not in their complete disappearance [47]. Nonetheless, modifications in alkaloid metabolism can impact primary metabolism. It has been shown, in fact, that suppression of benzophenanthridine alkaloid biosynthesis using antisense-BBE has the side effect of reducing the growth rate of the root cultures [41]. Similarly, the overexpression of 4-OMT in transgenic *Coptis japonica* plant resulted in an increase of berberine content, but also in a slower-growth phenotype [40].

Moreover, metabolic engineering of BIA biosynthetic pathway, besides the expected phenotype, often results in the accumulation of unknown/undesired metabolites [47] and this could be due to feedback regulation and metabolic channels.

Finally, the effects of genetic modification can be strongly different in individual plants. This could be due to genetic or physiological heterogeneity that strongly influences the amount of specific alkaloids. For example, silencing of codeinone reductase (CoR) in opium poppy (Scheme 7) resulted in the accumulation of reticuline, whose levels proved to vary in a wide range from plant to plant [45]. 

### 2.2. BIA Production in Microbial Systems: Bacteria and Yeast

#### 2.2.1. Bacterial Factories

Engineering microbial factories meet the quest for more sustainable and cheaper biotechnological processes for the production of alkaloids. As described in Section 2.1, alkaloids extraction from plants is characterized by low yields and requires many solvents; these problems are not completely overcome even when the engineering is directed towards the production of selected metabolites.

In recent years, many steps forward have been made in reconstituting BIA biosynthesis into microbial systems. Plant natural product biosynthesis in microbial hosts offers strategic advantages such as the chance of choosing the appropriate codon usage, finely tuning enzyme expression levels and timing, and manipulating host metabolism to increase precursor and co-factor concentrations [51].

Now, many pathways in BIA biosynthesis have been extensively characterized, allowing the opportunity of moving entire branches into microbial hosts such as *Escherichia coli* and *Saccharomyces cerevisiae*. Sometimes, the transplantation of a full metabolic pathway is not still enough to guarantee the desired results. For instance, it could be useful to increase the levels of the metabolic precursors of the desired BIAs reducing unwanted byproducts by metabolic flow modifications. This approach may lead to a reduction of the concentration of those enzymes associated with undesired microbial pathways.

**Scheme 8:**
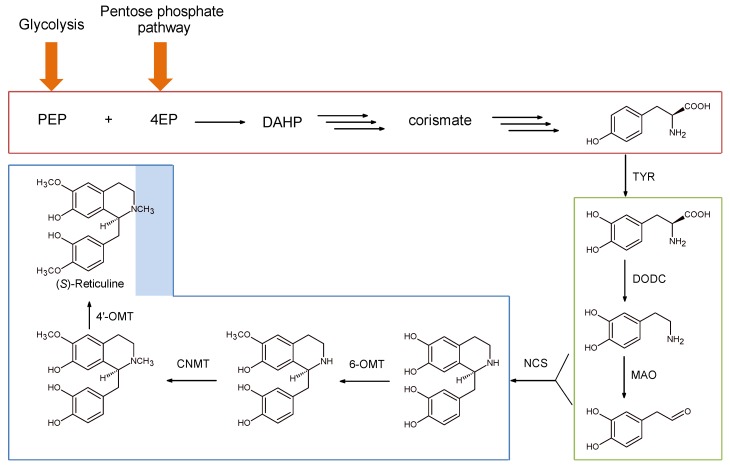
Metabolic pathways leading to the synthesis of (*S*)-reticuline in engineered *E. coli* strains. PEP: phosphoenolpyruvate; 4EP: eritrose-4-phosphate; DAHP: 3-deossi-arabinoeptulsonato-7-fosfato; DODC: l-DOPA decarboxylase; MAO: monoamine oxidase; 6-OMT: 6-*O*-methyltransferase; CNMT: coclurine-*N*-methyltransferase; 4′-OMT: 4′-*O*-methyltransferase.

A good example of synthetic pathway transplantation is represented by a work that can considered as a proof-of-concept study for a technology that allows the transfer of a great number of genes [52]. They developed a fermentative production in *E. coli* of (*S*)-reticuline, one of the main branch-point intermediates in the biosynthesis of different of BIAs (Scheme 7). Optimizing the production of (*S*)-reticuline could be useful for the synthesis of rare or non-natural BIAs to be tested for their potential physiological activities. The authors developed an *E. coli* strain overproducing tyrosine, the precursor of BIA backbone. This was accomplished by disrupting the tyrR gene (a repressor of aromatic amino acid biosynthesis), overexpressing genes of the shikimic acid pathway (feedback inhibition resistant aroG and tyrA), and introducing phosphoenolpyruvate synthetase (PEPS: ppsA) and transketolase (TKT: tktA) genes in order to hijack metabolic flow into the shikimic acid pathway (Scheme 8).

These bacteria were effectively able to produce larger amounts of tyrosine from simple carbon sources that can be used to generate dopamine and 4-hydroxyphenyl acetaldehyde (4-HPAA) whose condensation to (*S*)-norcolaurine represents the first committed step in BIA biosynthesis. l-DOPA was efficiently synthesized from l-tyrosine by tyrosinase form *Streptomyces castaneoglobisporus* (TYR; EC 1.14.18.1); 4-HPAA produced by tyrosine decarboxylation was catalyzed by monoamine oxidase from *Micrococcus luteus* (MAO; EC 1.4.3.4); (*S*)-norcoclaurine was generated by norcoclaurine synthase (NCS) from *Coptis japonica.* Finally, to convert (*S*)-norcoclaurine in (*S*)-reticuline three different plant methyltransferases (6-OMT, CNMT (coclurine-*N*-methyltransferase), and 4′-OMT) were also recombinantly expressed in this strain. Further optimization in the production yields (conversion efficiency 14-fold higher) was obtained by fine tuning the molar ratio of MAO and NCS that both compete for dopamine. This was realized cloning the genes into two plasmids with different copy number, under the control of IPTG (isopropil-β-d-1-tiogalattopiranoside) inducible T7 promoters.

In 2017, an improved system to produce large amounts of (*S*)-reticuline combining in vivo tetrahydropapaveroline production in *E. coli* with in vitro enzymatic synthesis of (*S*)-reticuline was developed using crude extracts of *E. coli* strains expressing methyltransferases [53]. The in vitro reaction catalyzed by methyltransferases was more efficient than in vivo fermentation system, due to the larger amounts of SAM that could be supplied. This experimental setting allows to obtain a further improvement in (*S*)-reticuline yields (593 mg/L isolated product).

#### 2.2.2. Yeast Factories

One of the limits of bacteria is that they are not suitable to stably express plant tailoring enzymes, such as the endomembrane-localized cytochrome P450s, which that are essential in many branches of BIA pathways. Eukaryotic hosts such as *S. cerevisiae* and *P. pastoris* can be used to overcome this limitation. They are very important model organisms for their capability to heterologously express complex, branched, multi-step biosynthetic pathways and challenging heterologous enzymes such as cytochromes P450. They allow the production of a number of different BIAs with different biotechnological engineering.

As an example, (*S*)-reticuline was produced in yeast starting from commercially available (*R,S*)-norlaudanosoline by using a combinations of enzymes both from plants (norcoclaurine 6-*O*-methyltransferase (6-OMT), coclaurine-*N*-methyltransferase (CNMT) and 3′-hydroxy-*N*-methylcoclaurine 4′-*O*-methyltransferase (4′-OMT)), and human (cytochrome P450 80B1 (CYP80B1)) [54]. 

**Scheme 9:**
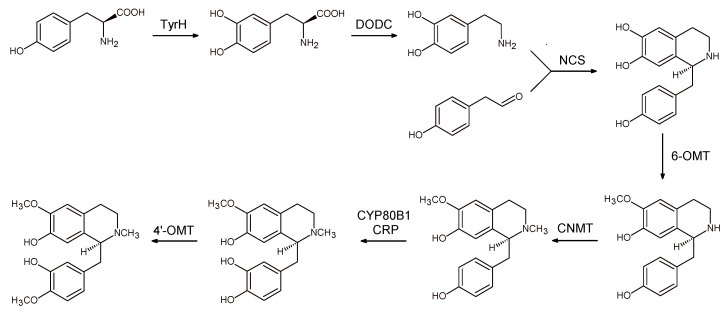
Reticuline biosynthesis in engineered yeast. TyrH: tyrosine hydroxylase; DODC: l-DOPA decarboxylase; NCS: norcolaurine synthase; CYP80B1: cytochrome P450; CRP: cytochrome P450 reductase; 6-OMT: 6-*O*-methyltransferase; CNMT: coclurine-*N*-methyltransferase; 4′-OMT: 4′-*O*-methyltransferase.

A further optimization of the process was achieved by combining enzymes from plants, yeast, mammals, and bacteria [55]. In this case, the first step was a modification of tyrosine metabolism to direct its flux through BIA pathway. Once the concentration of tyrosine was increased, heterologous expression of a mammalian tyrosine hydroxylase (TyrH), bacterial DOPA decarboxylase (DODC), and four enzymes associated with BH4 biosynthesis and recycling allowed to obtain 160 fold increase of (*S*)-noroclaurine, with fed dopamine without supplying exogenous tyrosine. As a final step, five enzymes from plant sources were incorporated (three methyltransferases, a cytochrome P450, and its reductase) to achieve the production of reticuline with a titer of 19.2 μg/L (Scheme 9).

Other authors engineered yeast strains to produce protoberberine alkaloid starting from (*R,S*)-norlaudanosoline adding more steps to the previously reported process [56]. They produced *S. cerevisiae* expressing seven heterologous enzymes including the flavin-dependent oxidase berberine bridge enzyme, the cytochrome P450 canadine synthase, and a cytochrome P450 reductase. As a result, canadine was obtained with a titer up to 1.8 mg/L.

**Scheme 10:**
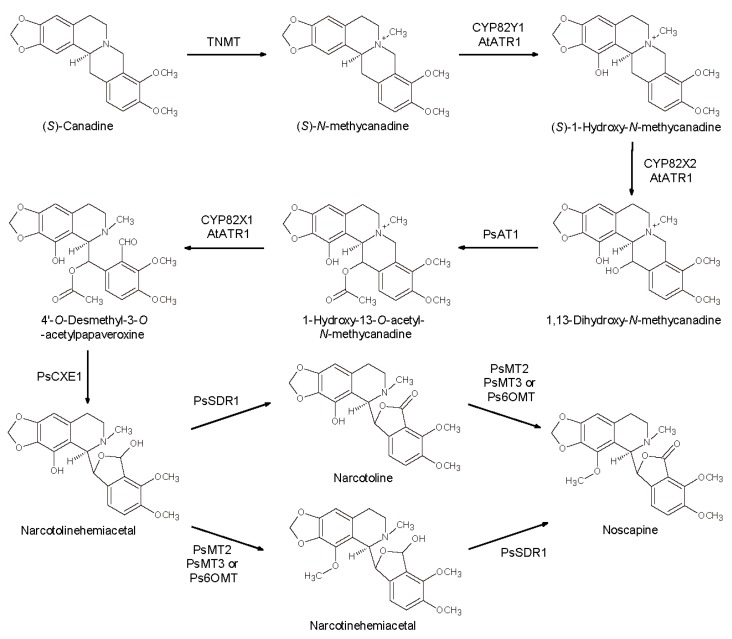
Noscapine biosynthesis in engineered yeast. TNMT: tetrahydroprotoberberine-*cis*-*N*-methyltransferase; CYP82Y1, CYP82X1, CYP82X2: cytochrome P450 monooxygenases; PsAT: acetyl transferase, PsCXE: carboxylesterase, PsSDR1: short-chain dehydrogenase/reductase; Ps6-OMT: methyltransferase; AtATR1: *Arabidopsis thaliana* cytochrome P450 reductase.

In 2016, the gene cluster leading to noscapine was reconstituted in *S. cerevisiae* achieving the microbial production of noscapine (titer of 1.64 ± 0.38 μM noscapine) and related intermediates [57]. Starting from canadine produced as described above, the authors developed yeast strains expressing different genes from *P. somniferum* (tetrahydroprotoberberine-*cis*-*N*-methyltransferase (PsTNMT), several cytochrome P450 monooxygenases (CYP82Y1, CYP82X1, CYP82X2), an acetyl transferase (PsAT1), a carboxylesterase (PsCXE1), a short-chain dehydrogenase/reductase (PsSDR1), a methyltransferase (Ps6-OMT)), and a cytochrome P450 reductase partner from *Arabidopsis thaliana* (AtATR1) (Scheme 10).

Based on the successful microbial production of (*S*)-reticuline, the main branch point in BIA biosynthesis, different research groups attempted to engineer the biosynthetic pathway leading to the alkaloid sanguinarine. One of these works proved the production of the metabolic intermediate stylopine from (*S*)-reticuline by using a *P. pastoris* expression system, characterized by high expression of recombinant proteins under the control of a strong inducible promoter [58]. Conversion of reticuline to stylopine was achieved by introducing berberine bridge enzyme, cheilanthifoline synthase (CYP719A5), and stylopine synthase (CYP719A2). The authors evaluated two experimental designs: in the first one these three genes were co-expressed in a single cell; in the second one each gene was expressed in a single cell and the three strains were co-cultured. This last approach was more efficient, as it resulted in the production of 150 nmol of stylopine.

Other authors were able to go further through this metabolic pathway. They transplanted a 10-gene plant pathway in *S. cerevisiae* leading to sanguinarine and dihydrosanguinarine synthesis [59] (see Scheme 7). Although only trace amounts of dihydrosanguinarine were obtained, this work can be reasonably considered the most complex plant alkaloid biosynthetic pathway ever reconstituted in yeast and shows the potential of engineering microbes for the synthesis of complex plant alkaloids.

Reconstruction of the sanguinarine branch of the BIA pathway in *S. cerevisiae* was also achieved [60]. The authors were able to improve cheilanthifoline, stylopine, *cis*-*N*-methylstylopine, protopine, and sanguinarine production (see Scheme 7) by expressing multiple plant cytochrome P450 enzymes, choosing the best performing isoenzymes and partner reductases, and paying particular attention to the best conditions for their expression and activity.

One of the most exciting challenges in microbial BIA synthesis is the fermentative production of the high value morphinan alkaloids in yeast, that are the strongest narcotic analgesics employed in pain treatment. Starting from 2008, researchers were able to create *S. cerevisiae* strains that can perform sections of the glucose-to-morphine pathway [54,59,61,62,63]. However, a single strain capable of executing the whole pathway is not yet available.

It is important to underline that the first committed step in the synthesis of morphine is the isomerization of (*S*)-reticuline to (*R*)-reticuline. A reticuline epimerase (a fusion between a cytochrome P450 and an aldo-keto reductase) was identified in opium poppy and in *Papaver bracteatum* [64]. This enzyme catalyzes the *S*-to-*R* epimerization of reticuline via 1,2-dehydroreticuline. Anyway, it cannot be excluded the existence of alternative pathways for the production of (*R*)-intermediates involving enzymes selective for the (*R*)-enantiomers from the beginning of the reticuline synthesis pathway [65].

The yeast-based plant pathway reconstitution is a valuable tool for understanding plant secondary metabolism and has the advantage of building strains for the production of intermediates that can be considered lead compounds for drug discovery. Nowadays, however, the isolated yields of BIAs produced with this approach are rather low and limited to a lab scale. 

## 3. Norcolaurine Synthase: The Gate of BIA Chirality

### 3.1. Chemo-Enzymatic Synthesis of BIAs

Among the different approaches for BIA production, in vitro biocatalysis is gaining more and more importance as it allows to produce complex THIQs in high stereoselectivities and under mild conditions. The first committed step in BIA biosynthesis is the Pictet-Spengler condensation of two simple molecules, dopamine and 4-HPAA, to yield the nitrogen heterocycle (*S*)-norcoclaurine (Scheme 7). This reaction is catalyzed by the enzyme norcolaurine synthase that introduces a chiral center that can be considered of paramount importance as it is needed for all the stereoselective enzymatic reactions characterizing the BIA biosynthetic pathways [66,67,68].

It is not surprising that many efforts have been directed towards the full biochemical characterization of NCS because it paves the ways to new, otherwise non-viable synthetic routes leading to benzylisoquinoline alkaloids. NCS activity was isolated in protein extracts of opium poppy (*Papaver somniferum*) and some other related species [66]. Soon afterwards, the enzyme form *Thalictrum flavum* was purified to homogeneity starting from cell culture [68] and then heterologously expressed in *E. coli* [67,69]. The first crystallographic structure of NCS from *T. flavum* in its complex with dopamine and the non-reactive substrate analogue 4-hydroxybenzaldehyde was obtained by our research group [70] allowing to understand NCS catalytic mechanism and kinetics [71]. Afterwards, it was demonstrated that a dopamine first mechanism governs the stereoselective production of (*S*)-norcoclaurine [72,73] and showed also that the kinetic behavior can explain the ineffectiveness of recombinant NCS in vivo systems. Nevertheless, NCS is a powerful tool for the in vitro asymmetric synthesis of (*S*)-norcolaurine and derivatives.

**Scheme 11:**
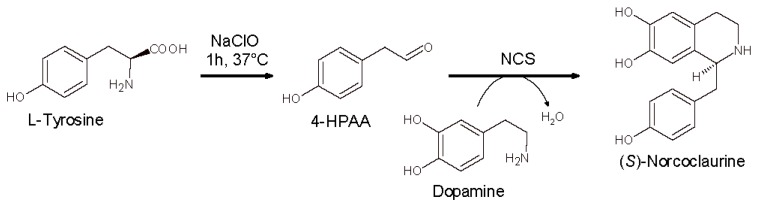
Chemo-enzymatic synthesis of (*S*)-norcolaurine starting from tyrosine and dopamine in the presence of norcoclaurine synthase (NCS).

Our group set up the first efficient, chemo-enzymatic synthesis of (*S*)-norcoclaurine using the recombinant NCS enzyme, starting from cheap substrates like tyrosine and dopamine in a one-pot, two-step process [74] (Scheme 11).

In the first step, 4-HPAA was generated by the oxidative decarboxylation of tyrosine in the presence of equimolar amounts of sodium hypochlorite. In the second step, dopamine and NCS were added to the reaction mixture in the presence of ascorbate to avoid catechol moiety oxidation. The process was carried out in phosphate buffer obtaining (*S*)-norcolaurinein 81% yield and 93% enantiomeric excess (*ee*).

Later investigations [75] showed that the phosphate buffer itself was able to catalyze the non stereoselective synthesis of norcolaurine, thus the choice of the buffer proved to be crucial for the stereoselectivity of the reaction. Phosphates are able to catalyze the reaction and show a very good tolerance for the aldehydes. The use of phosphate buffer represents an alternative to previously reported chemical strategies to synthesize simple tetrahydroisoquinoline alkaloids with mild reaction conditions. Nevertheless, this approach leads to racemic mixtures that need further resolution. To avoid background activity of phosphate in NCS catalyzed reaction, other buffers such as HEPES and MOPS should be used. This shrewdness allows for enantiopure products to be obtained. 

The reaction catalyzed by the NCS from different sources was tested for synthetic purposes as the specificity towards aldehyde substrates is quite relaxed, in contrast with the requirement for the amine substrate, which is limited to dopamine and few other dopamine analogues, highlighting the requirement of a meta-hydroxy moiety in dopamine [70,71,76,77,78].

In the light of the above, different research groups developed chemo-enzymatic strategies for the production of BIAs coupling a chemical approach for the aldehyde synthesis with the enzymatic activity of NCS.

One of these groups exploited the selectivity of *T. flavum* NCS towards fifteen chemically synthesized aldehydes including substituted phenylacetaldehydes, (hetero) aromatic substrates, aliphatic (hetero)cycles, and open chain aliphatic compounds [77]. These aldehydes, where not commercially available, were synthesized either by oxidation of the corresponding alcohols with Dess-Martin periodinane or by reduction of the corresponding Weinreb amide with lithium aluminium hydride (LAH). In the following step, these aldehydes were used as substrates in NCS catalyzed reaction. Although the synthesis was performed in lab scale and the enantiomeric excess of the reaction products was not investigated, this work paved the way to the chemo-enzymatic synthesis of non-natural THIQs. 

Another group, instead, investigated the substrate specificity of NCS from *Coptis japonica* (*Cj*NCS2) towards both the amine and aldehydic substrates [78]. In this work, more than ten structurally diverse aldehydes and amines were tested. The aryl and hetero aromatic acetaldehydes were synthesized from either the corresponding terminal alkenes via ozonolysis or the corresponding alcohols via a Parikh–Doering oxidation, while the ethylamines were produced from the corresponding phenylacetonitriles by means of standard reduction reactions. With this kind of approach the authors afforded (*S*)-THIQs, including the non-natural compounds in good to excellent yield (56–99%) and with an *ee* of 0.95%.

### 3.2. Fully Enzymatic Synthesis of Substituted Tetrahydroisoquinolines

A green improvement to the chemo-enzymatic methods for the production of BIAs described above is the employment of enzymes also for aldehyde production. Using enzymes in domino processes leads to the formation of highly complex compounds from cheap starting materials, often without intermediate isolation or functional group protection. In recent years, more and more attention has been devoted to these synthetic strategies.

**Scheme 12:**
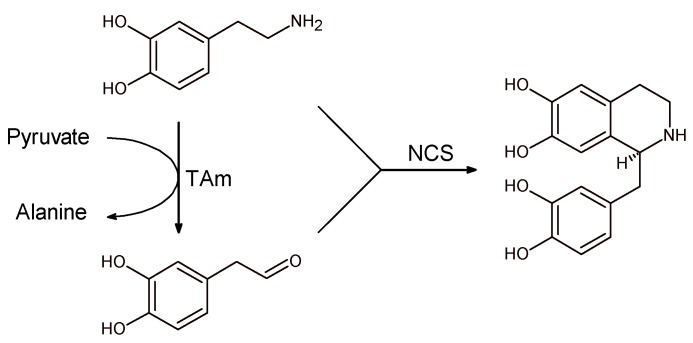
Fully enzymatic synthesis of norlaudanosoline involving transaminase (TAm).

The first attempt to perform a fully enzymatic synthesis of tetrahydroisoquinoline derivatives was achieved by coupling the activities of transaminase (TAm) and norcolaurine synthase in a one-pot, one-substrate “triangular” cascade (Scheme 12) [79].

Aldehyde species were generated in situ by transaminase (TAm) starting from dopamine that in its turn reacts with the newly synthesized aldehyde in the presence of NCS to give norlaudanosoline with good conversion and excellent enantioselectivity.

**Scheme 13:**
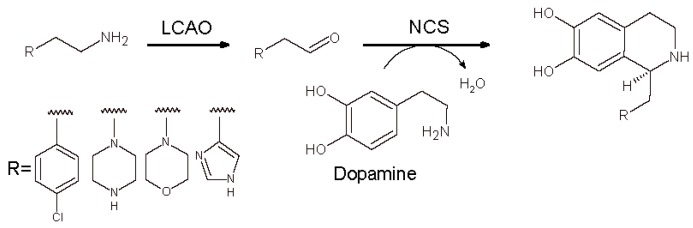
Fully enzymatic synthesis of substituted THIQs involving *Lathyrus cicera* amine oxidase (LCAO) and norcolaurine synthase (NCS).

More recently the aldehydes to be used in NCS catalyzed reaction were produced by oxidative deamination catalyzed by *Lathyrus cicera* diamine oxidase (LCAO) that shows a broad substrate specificity towards several ethyl amines [80,81]. Our group set up a fully enzymatic synthesis of new chiral THIQs in two steps starting from dopamine and four different amines by coupling the activity LCAO with *T. flavum* norcoclaurine synthase (*Tf*NCS) (Scheme 13). In this paper, four amines carrying morpholine or piperazine moieties were converted in the corresponding aldehydes and then used for the production of novel BIAs. All the reaction steps were performed in HEPES buffer.

The first step was optimized removing H_2_O_2_ produced by LCAO adding catalase. Before the second step, LCAO was inactivated with phenylhydrazine and then NCS and dopamine were added to the reaction pot. The newly synthesized BIAs were obtained in very good yields (>72%) and excellent enantioselectivity (*ee* > 99%).

**Scheme 14:**
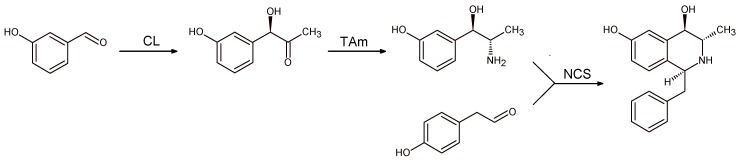
Fully enzymatic synthesis of 1,3,4-trisubstituted-THIQ involving carboligase (CL), transaminase (TAm), and norcolaurine synthase (NCS).

The first example of a three-step enzymatic cascade for the stereoselective synthesis of 1,3,4-trisubstituted-THIQ was reported in 2017 [82] (Scheme 14).

The first reaction step was catalyzed by carboligase (CL) that converted 3-hydroxybenzaldehyde and pyruvate in acyloin. In the second step, a transaminase (TAm) was used to form 2-amino-1-(3-hydroxyphenyl) propan-1-ol that in its turn reacted with phenylacethaldehyde in a NCS catalyzed Pictet-Spengler cyclization. The 1,3,4-trisubstituted-THIQ thus obtained was isolated excellent yield (92%).

**Scheme 15:**
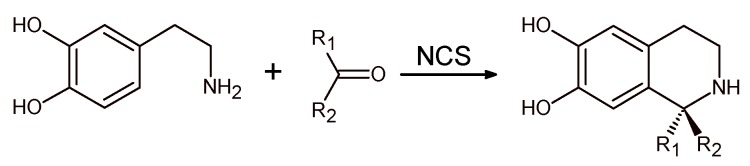
Norcolaurine synthase (NCS) catalyzed synthesis of chiral 1,10-disubstituted- and spiro-tetrahydroisoqionolines.

In the same year, *Tf*NCS variants with improved ketone tolerance that were able to accept non-aldehyde carbonyl substrates were identified [83]. These enzymes could accept a number of unactivated ketones for the synthesis of new chiral 1,10-disubstituted- and spiro-tetrahydroisoqionolines (Scheme 15). 

The (chemo)-enzymatic methods here reported for the synthesis of natural and non-natural BIAs demonstrate the great potential of in vitro biocatalysis for the formation of complex chiral compounds. The main advantage of these domino processes is that they do not require intermediate isolation or functional group protection strategies. Moreover, all these reactions are performed in aqueous media and exhibit high atom economy.

However, the critical step is represented by the lab scale production that needs to be improved at least to gram scale. This improvement can be in principle achieved by finely tuning the enzyme activity and/or by increasing its stability and recycling. In fact, many enzymes lack of long-term stability under the experimental conditions and it is also difficult to recover and recycle them in order to make the overall process economically and environmentally advantageous. Enzyme immobilization can be a useful tool for enhancing stability, reducing costs and modulating catalytic properties.

## Abbreviations

BIAs	Benzylisoquinoline alkaloids
THIQs	Tetrahydroisoquinolines
4-HPAA	4-hydroxyphenylacethaldehyde
NCS	Norcoclaurine synthase
VIGS	Virus-induced gene silencing
CRISPR	Clustered regularly interspaced short palindromic repeats
OMT	*O*-Methyltransferase
CMNT	Coclurine-*N*-methyltransferase
TNMT	Tetrahydroprotoberberine-*cis*-*N*-methyltransferase
BBE	Berberine bridge enzyme
DODC	l-DOPA decarboxylase
MAO	Monoamine oxidase
LCAO	*Lathyrus cicera* amine oxidase
CYP	Cytochrome P450 monooxygenases
TAm	Transaminase
